# Review of Studies Comparing Panretinal Photocoagulation and Anti-Vascular Endothelial Growth Factor Therapy in the Treatment of Proliferative Diabetic Retinopathy

**DOI:** 10.7759/cureus.22471

**Published:** 2022-02-21

**Authors:** Thiruvarasu Gunasekaran, Yuarrani Gunasekaran, Pua Tze Hui

**Affiliations:** 1 Ophthalmology, Hospital Seri Manjung, Sitiawan, MYS; 2 Ophthalmology, Hospital Bintulu, Bintulu, MYS; 3 Family Medicine, Klinik Pantai Remis, Perak, MYS

**Keywords:** diabetes mellitus, anti vegf, laser prp, diabetic retinopathy, pdr

## Abstract

Diabetic retinopathy (DR) is among the leading causes of blindness at the global level. A review of studies between 2015 and 2018 found that about 1.7% of the general population with any type of diabetes mellitus suffered from proliferative diabetic retinopathy (PDR). Since the 1960s, panretinal photocoagulation (PRP) has been the mainstay of treatment for PDR. During this period, PRP has been credited with a significant degree of success and a relatively low complication rate. However, the advent of anti-vascular endothelial growth factor (anti-VEGF) therapy with the beginning of the new millennium provided a treatment modality that was noninferior to PRP. A decade-long period of comparisons and debates between these two treatment modalities repeatedly favored anti-VEGF over PRP, as studies demonstrated that the former provided potentially superior outcomes to PRP. The aim of this review is to briefly discuss and compare the relevant studies and evidence supporting these two treatments.

## Introduction and background

Diabetes mellitus (DM) is a chronic metabolic disease that leads to serious damage to various organs, including the eyes [1]. Both the number of cases and the prevalence of DM have steadily increased over the past few decades. The World Health Organization (WHO) estimates that about 422 million people worldwide were living with DM in 2014, compared with 171 million in 2000 [2], particularly in low- and middle-income countries and 1.6 million deaths are directly attributed to DM each year [3-5]. The global diabetes prevalence for 2019 is estimated to be 463 million people and prediction says this number will increase by 51% in 2045 [6].

Diabetic retinopathy (DR) is a major ocular complication associated with DM and is one of the leading causes of acquired vision loss [2]. From 2.6% to 4.8% of global cases of blindness can be attributed to DR [5, 7-8], and it is estimated that more than 75% of patients who have DM for more than 20 years will have some form of retinopathy [5]. The statistics show that in patients who have had insulin-dependent DM for at least 20 years, proliferative diabetic retinopathy (PDR) is likely to be a pre-existing condition for 60% of the patients [5]. Of those who have had DM for 30 years or more, about 12% are blind [5]. Pooled data from 35 studies from 1980 to 2008 on 22,896 individuals with DM provided global estimates of the prevalence of DR and vision-threatening diabetic retinopathy (VTDR) of 34.6% and 10.2%, respectively. However, a recent systematic review provided an updated estimate of the prevalence of DR and VTDR of 22.27% and 6.17%, respectively [9]. These numbers were attributed to a combination of factors including growing public interest and awareness regarding DM in Asia, leading to a higher number of screenings and earlier diagnosis of DM in high-risk populations in many Asian countries.

Diabetic retinopathy can be classified into two broad categories, nonproliferative DR (NPDR) and PDR, with the latter being a vision-threatening condition [10]. NPDR is clinically associated with microaneurysms, increased vessel permeability, retinal hemorrhages, and vessel occlusion [10]. The distinguishing feature of PDR, on the other hand, is neovascularization [10]. For the majority of patients with PDR, the primary treatment of choice in the past has been panretinal photocoagulation (PRP). Laser photocoagulation is a destructive therapy because it destroys photoreceptors and other metabolically active cells of the retinal pigment epithelium (RPE), simultaneously lowering the oxygen consumption of these cells [11]. In return, vascular endothelial growth factor (VEGF) which is responsible for reinstating oxygen levels in cells is down-regulated, resulting in regression of PDR [12]. However, the destroyed retinal cells contribute to the deterioration of the peripheral field of vision and night vision. The downside of PRP led scientists to search for an angiogenic factor which was discovered in 1989 when Ferrara isolated and cloned VEGF [13]. The discovery of VEGF changed the way DR has been treated since the advent of anti-VEGF [14] and has been shown to provide better outcomes in several studies. However, the use of anti-VEGF has limitations due to factors such as poor compliance, financial burden, frequent need for injections, side effects, and uncertain end results, making PRP still relevant as an adjuvant treatment. The aim of this review is to compare recent studies supporting these two treatment modalities.

## Review

Methods

A comprehensive literature search was conducted using different Internet-based search engines such as Google Scholar and bibliographic databases (PubMed, PubMed Central, MEDLINE, Medknow, Scopus, Web of Science) from August 2020 to January 2021. This integrative search was conducted using the keywords “PDR,” “anti-VEGF,” “PRP,” “diabetic retinopathy,” and “diabetes”. The search was limited to articles published in the English language and those published between the years 2000 and 2021. Articles with duplicate data were excluded, and a total of 103 articles were initially identified after being screened and assessed by the titles and abstracts. After a vigorous assessment, we included data from 57 articles for this review process. Bibliographic management was done using the software “EndNote”.

Clinical trials comparing anti-VEGF monotherapy and PRP for the treatment of PDR

Diabetic Retinopathy Clinical Research Protocol S (2012-2015)

The primary objective of Protocol S [15] (ClinicalTrials.gov Identifier: NCT01489189) was to compare the safety and efficacy of PRP with that of intravitreal ranibizumab injections for the treatment of PDR. A total of 305 adults with PDR were enrolled in this randomized clinical trial, with a total of 394 study eyes. Subjects with best-corrected visual acuity (BCVA) letter scores of 24 or higher were randomly allocated into two groups. In one group, the study eyes received a 0.5-mg intravitreal injection of ranibizumab at baseline and every four weeks for 12 weeks. Injections at months 4, 5, 6, and thereafter were administered according to the clinician’s interpretation of neovascularization. PRP was allowed for treatment failures. In the other group, PRP was initiated at baseline and completed in one to three visits. Additional PRP was given if neovascularization showed signs of worsening. Eyes with baseline diabetic macular edema (DME) were given intravitreal ranibizumab injections at initiation and thereafter, at the investigator’s discretion, in both groups. This treatment protocol resulted in a median of seven injections in eyes without DME at baseline and nine injections in eyes with DME at baseline at year one of the study.

At two years, 97% of study eyes in the ranibizumab group had received intravitreal injections. Of these, 6% needed rescue PRP. Accordingly, 98% of eyes in the PRP group completed PRP, with 45% needing additional PRP. In the PRP group, 35% of those with DME at baseline received intravitreal injections, and another 18% received intravitreal injections for DME before two years. At two years, the improvement in mean visual acuity (VA) letter score from baseline was +2.8 in the ranibizumab group and +0.2 in the PRP group, with a significant difference between the groups noted at one year. A similar outcome was seen for the visual field (mean change combining 30-2 and 60-4 total point scores) with changes from baseline of −23 dB [SD, 410 dB] versus −422 dB [SD, 518 dB], in the ranibizumab versus PRP group, respectively. In addition, patients in the ranibizumab group showed a significant reduction in central subfield thickness (CST) compared with the PRP group at the end of two years, in both groups with and without baseline DME. The percentage of eyes with regressed neovascularization was similar in the two groups: 35% in the ranibizumab group and 30% in the PRP group. In addition, 48% of eyes in the ranibizumab group showed an improvement of two or more steps in the diabetic retinopathy severity scale (DRSS).

The study concluded that intravitreal ranibizumab met the noninferiority outcome of VA change at two years when compared with the PRP group for treatment of PDR. In addition, patients in the ranibizumab group were found to have a less peripheral visual loss, fewer vitrectomies performed, fewer eyes with DME with visual impairment, and better VA when evaluated at two years.

Five-Year Outcome for Protocol S (2012-2018)

In July 2018, the five-year data for Protocol S [16] were published, based on 240 study eyes in the ranibizumab and PRP groups. No major modifications of the treatment had been made since the original two-year trial. The study concluded that VA at the five-year visit was improved from baseline, resulting in a mean VA of 20/25 in both groups compared with 20/32 at baseline. In addition, 46% of study eyes in the ranibizumab group showed improvement from baseline by two or more steps in the DRSS scale. The percentage of eyes with neovascularization at the end of the five-year study was similar in the two groups, ranging from 29% to 30%. It was also reported that the study eyes in the ranibizumab group were less prone to developing DME and that those which had DME improved from baseline. The probability of developing vision-impairing DME by five years was 22% in the ranibizumab group, compared with 38% in the PRP group. The study reported a higher rate of retinal detachment in the PRP group than in the ranibizumab group, 18% versus 7%, although most of these cases were not vision-threatening.

The mean number of required injections was reduced each year from 7.1 in year 1 to 2.9 in year 5. By year 5, 37% of eyes in the ranibizumab group did not require any injections. The mean cumulative number of injections over the five years was 19.2. Fourteen percent of eyes in the ranibizumab group needed PRP throughout the entire five years. In comparison, of the total of 203 eyes that received PRP at baseline in the PRP group, 51% received at least one additional PRP, and 89% of these eyes needed PRP before two years. Among the eyes in the PRP group that completed five years, 58% needed at least one anti-VEGF injection for DME. The mean cumulative number of injections over the five years was 5.4. The authors recommended caution in the interpretation of the results, considering that only 60% of the initial participants completed the five-year trial.

CLARITY Study (2014-2015)

This randomized Phase 2B trial [17] was conducted on 232 patients in the United Kingdom to study the clinical efficacy of intravitreal aflibercept versus PRP in patients with PDR. This trial was considered a cleaner study than Protocol S, with tighter exclusion criteria, excluding patients with DME at baseline. Aflibercept was used in the anti-VEGF control groups. Baseline vision in both the anti-VEGF and the PRP groups was equivalent to Snellen 6/24 or greater. Patients in the aflibercept group were given three loading doses of intravitreal injections every four weeks. Further treatment at weeks 12 and 16 is determined by both regression and reactivation of retinal neovascularization on clinical examination. The PRP treated eyes were given PRP at baseline in two weekly sessions. They were reassessed at week 12 for persistent new vessels and every eight weeks thereafter.

The study found aflibercept superior to PRP. The mean change in BCVA was −3.0 letters (SE 0.7) for PRP and 1.1 letters (SE 0.6) for aflibercept (difference, 3.9; 95% CI, 2.3-5.6; p<0.0001). Patients in the aflibercept group had significantly higher rates of regression of new retinal vessels at 52 weeks than those in the PRP group, with a difference between groups of 30%. Only 78% of patients in the aflibercept group remained at PDR in contrast to 90% in the PRP group. Eyes treated with PRP had a higher incidence of macular edema and VH as well: 29% versus 11% and 18% versus 9%, respectively. Satisfaction scores showed that patients preferred aflibercept to PRP. At 52 weeks, the mean number of injections received by patients in the aflibercept group was 4.4, including three loading doses, whereas 65% of the patients in the PRP group required supplemental PRP from week 12, with a mean of 1.17. Of the 114 patients receiving PRP treatment, 31% required more than three sessions. The short period of follow-up (52 weeks) was a limitation of this study.

Clinical trials comparing anti-VEGF combination therapy and PRP in treatment of PDR

PRIDE Study (2012-2017)

The PRIDE study [18] was a Phase 2 randomized clinical trial comparing the efficacy of ranibizumab, with or without the combination of PRP, versus PRP alone in treating PDR. This study differed from Protocol S by the addition of a combination group and exclusion of eyes with DME. A total of 106 patients were randomly allocated to treatment.

The ranibizumab group received monthly injections for three months, after which subsequent injections were based on clinical assessment. The PRP group received from 1,200 to 1,600 laser shots between baseline and month 3; further shots were repeated at the investigator’s discretion. Patients in the combination group received both types of treatment according to the prespecified algorithm. Patients in the ranibizumab group received rescue PRP from the second injection onward if there was evidence of progression of neovascularization.

At the end of the study at month 12, the outcome favored the ranibizumab group. Eyes receiving ranibizumab showed a significant reduction in the mean area of neovascularization from 9.39 ± 15.41 to 2.70 ± 4.11 mm². The least square (LS) mean difference in neovascularization area between the ranibizumab monotherapy and the PRP groups (−2.83 mm2) was also significant. Complete regression of neovascularization leakage at month 12 was observed in 27.6%, 7.7%, and 17.9% of patients in the ranibizumab, PRP, and combination groups, respectively. The study reported an increase in BCVA from 83.3 ± 7.4 letters at baseline to 84.4 ± 8.6 letters at month 12 and a reduction in CST of 6.0 ± 15.1 μm in the ranibizumab group. Data from the Early Treatment Diabetic Retinopathy Study (ETDRS) severity scales showed no significant differences between the treatment groups. At the end of the study, the PRP group had the lowest percentage of NPDR patients at 4%, whereas the ranibizumab and combination groups had the highest percentages of NPDR patients, at 14% each. Five patients needed vitrectomy during the entire study, none of whom were in the ranibizumab group. Eleven percent of patients in the ranibizumab group received one rescue PRP during the trial period.

The results for the ranibizumab group were plausible, considering that the median number of injections over the 12-month period was only five, in contrast with Protocol S, which resulted in a median of seven to nine injections in year one. However, the investigators concluded that there was possible undertreatment in both anti-VEGF groups since the rapid improvements that were seen at month 3 were not sustained to the end of the study. These improvements included change in CST, change in total neovascularization, and improvement in the ETDRS severity scale. The investigators also pointed out the short duration and small sample size as limitations of this study.

Proteus Study (2014-2017)

This randomized Phase 2/3 trial [19] aimed to study the effect of PRP plus intravitreal ranibizumab versus PRP alone on the regression of neovascularization in PDR patients. A total of 77 patients completed the study. The exclusion criteria included recent PRP or intravitreal anti-VEGF injection within three months before study enrolment and neovascularization at baseline. The study included only eyes at high risk for PDR and without DME at baseline.

Patients were randomly assigned to receive PRP plus intravitreal injection (combination group) or PRP alone (monotherapy group). The combination group received three-monthly intravitreal injections combined with one to three sessions of PRP. Treatment from months 3 to 11 was at the investigator’s discretion and included one intravitreal injection and one PRP session during each retreatment period. The PRP monotherapy group was treated with complete PRP sessions between day 1 and month 2. Treatment thereafter until month 11 was at the investigator’s discretion. The median number of injections and PRP treatments in the combination group was four and three, respectively. In the PRP monotherapy group, the median number of PRP treatments was five.

At month 12, 92.7% of patients in the combination group versus 70.5% in the PRP monotherapy group had a total reduction of neovascularization. The rates of reduction of isolated neovascularization at disk (NVD) and neovascularization elsewhere (NVE) were also higher in the combination group; the difference between the groups in NVE reduction was statistically significant. The rate of complete neovascularization regression was 43.9% in the combination group versus 25% in the monotherapy group; this regression was seen much earlier in the combination group. However, 67% of the eyes from the combination group that achieved total regression of neovascularization had a recurrence of new vessels. In addition, the combination group had better improvement in mean BCVA than the monotherapy group, although the difference was not statistically significant. The mean final CST was lower in the combination group. Rescue treatment was needed in seven patients in the monotherapy group and one patient in the combination group.

The study concluded that patients with PDR had more regression of neovascularization when treated with PRP combined with intravitreal injection rather than with PRP alone. The short duration and the small sample size were limitations of the study.

RELATION Study (2010-2011)

This Phase 3b study [20] was designed to compare the efficacy of the combination of intravitreal ranibizumab and laser PRP versus PRP monotherapy in NPDR and PDR patients with DME. The study was prematurely ended when ranibizumab received approval for the treatment of DME shortly after initiation of the study, and further randomization of patients to monotherapy was considered unethical.

A total of 128 patients were randomly assigned to the combination and monotherapy groups at a 2:1 ratio, respectively. Patients in the combination group received four intravitreal injections from baseline to month 3. Both groups received laser treatment as standard at baseline and if needed at month 3 at the investigator’s discretion. Retreatment from month 4 to study end was based on the modified pro re nata (PRN) regimen for both groups. The criterion for retreatment was worsening of DME, judged on the basis of BCVA and central retinal thickness.

Although the objective of the study was to evaluate DME as the primary targeted condition using different treatment regimens, the study did report that in patients with PDR at baseline, the change in BCVA from baseline to study end favored the combination group, with LS mean difference of 14.7 (−7.93; 37.33), (p = 0.1077). Other results favoring the combination group include a significant decrease in CST and total retinal volume from baseline to month 4 and study end, as well as a significant decrease in inner retinal thickness, in PDR and NPDR patients. The mean number of injections received by the combination group during the entire study was 5.0. The main limitation of this study was the small sample size due to the premature termination of the study.

Other trials

Mirshahi and colleagues [21] conducted a controlled clinical trial on 40 high-risk PDR patients to evaluate the effect of a single intravitreal injection of bevacizumab on laser-treated eyes. All 80 eyes underwent standard PRP treatment and a single anti-VEGF injection in one eye, while the contralateral eyes received sham injections and laser treatment. The study found that 87.5% of anti-VEGF-injected eyes had complete regression at week 6, as opposed to 25% in the sham group. However, the difference between the groups in partial and complete regression diminished at week 16. Hemoglobin A1C was the only factor correlated with recurrence of PDR.

Tonello and colleagues [22] conducted the IBeHi study with a small number of eyes using leakage area and BCVA as outcome measures. Thirty eyes with high-risk PDR were randomly assigned to receive PRP alone or PRP plus bevacizumab. These eyes had no previous history of laser treatment. Although there was no improvement in BCVA, the group receiving PRP plus bevacizumab had a significant reduction in the total area of leakage compared with the group receiving PRP alone, with the most substantial reduction recorded in the first four weeks from baseline. An increase of leakage between weeks 9 and 16 suggests that additional anti-VEGF injection may have been needed.

Zhou and colleagues [23] conducted a similar study by randomly assigning 36 eyes to receive PRP alone or PRP plus bevacizumab. Similar to the IBeHi study, Zhou reported a marked reduction in the leakage area in the group receiving PRP plus bevacizumab as well as improvement in BCVA at the end of 24 weeks. The investigators attributed the improvement in BCVA to clearing of pre-existing VH and improvement in DME. In addition, a significant decrease in CST was seen in the group receiving PRP plus bevacizumab.

Ongoing trials

There are various ongoing clinical trials that aim to address the effect of both types of treatment on PDR. One such study is a Phase 3 trial that aims to evaluate whether brolucizumab is noninferior to PRP for the treatment of PDR (ClinicalTrials.gov Identifier: NCT04278417). Brolucizumab is a new-generation anti-VEGF drug that was recently approved by the US Food and Drug Administration (FDA) for wet age-related macular degeneration (AMD). It is a humanized single-chain antibody fragment VEGF inhibitor with very low molecular weight. The study, which is predicted to be completed in 2024, is currently recruiting 706 participants with types 1 and 2 DM. The exclusion criteria include previous PRP treatment, recent intravitreal anti-VEGF injection, and the presence of DME. The study’s primary outcome is the change in BCVA. The secondary outcome measures include presence of PDR and center-involved, DME at the end of the study, change in ETDRS scale, presence of vision-threatening complications, and change in area under the curve for BCVA. As studies have shown that brolucizumab predisposes patients to intraocular inflammation, a high index of suspicion is required during each assessment [24].

Another new drug is conbercept, a recombinant fusion protein VEGF inhibitor that is used in China for the treatment of AMD. CONTINENT (ClinicalTrials.gov Identifier: NCT02911311) is a study that aims to identify the efficacy of conbercept and PRP in the treatment of PDR. The primary outcome is the change in BCVA. The secondary outcomes are the regression pattern of new vessels and change in the visual field, among others. The recruitment status of this trial is unknown as of today.

Protocol W (ClinicalTrials.gov Identifier: NCT02634333) is another ongoing trial that is primarily designed to compare eyes receiving early treatment with anti-VEGF versus eyes that are observed initially and treated only when PDR or DME develops. The experimental group’s treatment protocol differs slightly from other trials, as anti-VEGF injections are given at one, two, and four months and then every four months. The treated eyes will be watched for any neovascularization, center-involved DME, or outcomes of PDR. Some of the inclusion and exclusion criteria are still being evaluated.

There are also ongoing studies that evaluate the primary outcome using different measures. For example, the PROPER study (ClinicalTrials.gov Identifier: NCT04674254) is designed to assess changes in foveal avascular zone area and vascular density of the retinal capillary plexuses in PDR eyes treated with anti-VEGF versus standard PRP. The study’s secondary outcomes include the change in central macular thickness, change in macular sensitivity, and change in new vessels. The KIA-ProRet trial (ClinicalTrials.gov Identifier: NCT00776763), on the other hand, measures growth factors and other cytokines as a primary outcome in anti-VEGF-treated PDR eyes. These studies will allow us to better understand the way anti-VEGFs work to improve vision in PDR patients. Both studies are using bevacizumab as their anti-VEGF drug.

Some studies are still being conducted using widely established anti-VEGF drugs, such as bevacizumab (ClinicalTrials.gov Identifier: NCT02705274), ranibizumab (ClinicalTrials.gov Identifier: NCT01280929), and aflibercept (ClinicalTrials.gov Identifier: NCT02151695), compared with standard PRP for treatment of PDR. Most of these studies evaluate the efficacy of anti-VEGF drugs based on regression of retinal neovascularization. There is also a study using the first FDA-approved drug for wet AMD, pegaptanib (ClinicalTrials.gov Identifier: NCT01281098).

Discussion

Anti-VEGF medications have proved to be an important breakthrough in the treatment of PDR. The studies discussed above have demonstrated that anti-VEGF can be used as an adjunct to PRP, if not as a replacement. Almost all the outcomes favor anti-VEGF, with improved gains in VA. Studies such as Protocol S demonstrated that anti-VEGF alone is noninferior to PRP, whereas some studies, such as CLARITY, demonstrated superior visual outcome compared with the 60-year-old PRP. In addition, eyes treated with anti-VEGF had significant reduction in CST, milder degrees of vision-impairing DME, regression of neovascularization, and subsequently less neovascular leakage. Visual field loss was also less than in eyes treated with destructive PRP. Another notable advantage of anti-VEGF is higher regression from PDR to NPDR in treated eyes.

These are important indicators to start anti-VEGF treatment early in severe NPDR, especially as an alternative to PRP, because anti-VEGFs have the potential to reduce the severity of DR, especially when DME coexists. This was demonstrated in studies of other eye diseases, such as the PANORAMA [25], VIVID and VISTA [26], and RISE and RIDE [27-28] studies. The RISE and RIDE study, for instance, stated that proliferative disease appeared to have a less robust response to anti-VEGF than did advanced or less advanced retinopathies, suggesting a shift from the usual paradigm of starting anti-VEGF only with the recognition of PDR. In addition, early treatment with anti-VEGF decreases the need for additional treatment and reduces the energy required for PRP, if needed [29]. In cases where the ophthalmologist decides to cross over from PRP to anti-VEGF halfway into the initiation of treatment, studies have shown that the outcomes of delayed crossover treatment are not as promising as the outcomes of prompt treatment with anti-VEGF once a diagnosis is established [30-31]. This has been attributed to irreversible structural damage caused by earlier PRP or a slow chronic phase of DME [30].

The question remains, however, whether to choose anti-VEGF over PRP. The decision has been a constant dilemma for ophthalmologists in developing countries. Cost, compliance, and practicality have to be considered when choosing the best treatment for the patient [32]. There are some disadvantages to choosing anti-VEGF over PRP. One of the few advantages of PRP is its lower cost, especially in some countries in Asia, where free PRP treatment is offered to patients in the form of subsidy in the same health care facilities where anti-VEGF medications are required to be purchased at the patient’s own expense. A Markov-style modeling of the costs of primary treatment of PDR found that PRP is still the less expensive treatment, regardless of the practice setting, yielding a cost per quality-adjusted life year (QALY) 58%-61% lower than that of intravitreal ranibizumab [33]. This study also concluded that both PRP and intravitreal ranibizumab initial treatments are still well below the QALY value considered acceptable by health policymakers ($50,000-$100,000), but proposed further cost increments for intravitreal injections after two years. Another preplanned secondary analysis using the two-year data from Protocol S suggested that PRP is more cost-effective than ranibizumab for patients with PDR without baseline vision-impairing DME [34]. Both of these studies did, however, explore the vision-related quality of life and dual advantage of anti-VEGF in concomitant treatment of DME and PDR.

One contributing factor that adds to the high cost of anti-VEGF treatment is its more intensive follow-up. PRP can be completed within a few visits, and once the condition is stabilized, there is rarely a need for additional treatment or frequent follow-ups. Furthermore, the long-term effectiveness of PRP has been established. A study of DR conducted by the Bascom Palmer Eye Institute found that PRP provided good results for at least 15 years, with only 5% of argon-treated and 3% of xenon-treated eyes receiving additional laser treatments [35]. In contrast, anti-VEGF treatment is intensive and requires frequent monitoring.

The duration and number of anti-VEGF injections are often unknown, and at times, the extended but indefinite treatment regimen can exhaust patients. This may place an increased burden on health care services and carers as well. For comparison, in the Protocol S study, the average interval from completion of the first full PRP to the next PRP for patients in the PRP group was 210 days or about seven months. In contrast, in the anti-VEGF group, the median number of anti-VEGF injections required during the first year was seven to nine at bi-monthly intervals. Furthermore, the duration of the period of injections is uncertain and may extend over longer periods. There is also the added risk of severe progression of the disease to tractional retinal detachment (TRD) and irreversible visual loss in patients who are noncompliant to follow-up. In Protocol S, only 60% of the patients were evaluated at the five-year outcome.

Assessment of eyes following treatment for PDR involves not only VA outcomes but, more importantly, complete regression of neovascularization and the rate of its recurrence. Although there was no significant difference in VA between PRP- and anti-VEGF-treated eyes in year 5 of Protocol S, active neovascularization was seen in 29% of eyes treated with ranibizumab. The mean number of injections required up to five years was 19, with a mean of 2.9 injections yearly during years 3 to 5. It is not known how long the injections need to be continued. The dropout rate of 40% is also a concern. Recurrence of new vessels after complete regression in eyes treated with anti-VEGF injections was observed in the Proteus study, in which only the ranibizumab-treated eyes had recidivism. In a clinic with a high burden of cases and with different levels of doctors performing evaluations, there is a risk of overlooking the progression in some patients.

In addition, the risk of arterial thromboembolic events (ATEs) following repeated intravitreal anti-VEGF injections has been established. The five-year data from Protocol S showed an increased trend toward systemic ATEs among participants assigned to ranibizumab [36]. The CLARITY study reported that ATEs were twice as frequent in the aflibercept group as in the PRP group. Other reported ocular adverse events include worsening of pre-existing TRD [37-39], caused by the intensive contractive effect of fibrous components and tissues by anti-VEGF. Risks of endophthalmitis, macular hole, and intraocular pressure (IOP) elevation have also been reported [40].

Another important factor to consider in the treatment of PDR is the patient’s choice in the selection of the modality of treatment. Although Protocol S showed that anti-VEGF treatment helped patients in some work- and driving-related tasks, there were no real differences noted in patient-centered outcomes between the two treatment methods [41-42]. The CLARITY study, which showed a preference for anti-VEGFs according to patients’ satisfaction scores, was evaluated early because it was conducted for only 52 weeks.

The studies discussed here have limitations. Loss to follow-up [43], short duration, unequal sample sizes, small sample sizes, complications affecting the progress of the studies, and flawed inclusion criteria are some of the limitations that need to be addressed. The relative superiority of different anti-VEGF drugs is also not discussed. Additional improved studies are needed, and the long-term outcomes of anti-VEGF treatment of PDR need to be further evaluated with larger, controlled trials.

Although anti-VEGF has better visual outcomes in PDR patients, patient factors such as socioeconomic status, systemic health, low health literacy, and transportation as well as socio-economic issues have to be taken into consideration when choosing the best treatment for PDR, thus making PRP still a relevant option. At present, PRP remains a proven treatment for PDR without DME, but anti-VEGF medications have clear advantages for treating DME along with PDR [44] and are beneficial for regression of neovascularization in NVG [45-47] and DR [48-50], in addition to substituting for PRP where laser treatments are not feasible. Other studies have indicated that anti-VEGFs can also be used to ease surgeries, as an accessory either to cataract surgeries [51-53] or to pars plana vitrectomy for nonclearing VH [37] and TRD [54-57]. Studies have shown that the effect of anti-VEGF is transient, but the improvements that anti-VEGF brings to PDR patients, although short-lived, are too remarkable to be neglected.

## Conclusions

Anti-VEGF drugs have proven their potential and managed to address the weaknesses that PRP had, in the treatment of PDR. However, laser photocoagulation has its own advantages which explain why the traditional method worked for many years. In summary, there are pros and cons to the use of both treatment modalities, and the choice should be individualized based on various ocular and patient factors. Combining laser PRP or even subthreshold laser PRP with anti-VEGF should be an option that needs further studies as well. The results of ongoing trials will hopefully provide a more promising approach to the treatment of PDR.
